# Retraction: Morphophysiological changes in the splenic extracellular matrix of *Leishmania infantum*-naturally infected dogs is associated with alterations in lymphoid niches and the CD4+ T cell frequency in spleens

**DOI:** 10.1371/journal.pntd.0010225

**Published:** 2022-02-16

**Authors:** The PLOS Neglected Tropical Diseases Editors

After this article [1] was published, concerns were raised about regions of similarity within the microscopy images in Fig 2.

Specifically:

- There appear to be similarities between adjacent regions within the bottom right corner in Fig 2A.
- There appear to be similarities between several regions within Fig 2C.
- There appear to be similarities between several regions within Fig 2F.
- There appear to be similarities between several regions within Fig 2G.

A set of underlying images for panels A-M of Fig 2 were provided.

Editorial assessment of the provided data identified additional concerns that some features shown in the underlying images for Fig 2E and 2H do not appear to be present in the corresponding published figure panels. In response to queries about this figure, the corresponding author has indicated that panels within Fig 2 were altered to improve their aesthetics and to cover artifacts, and that although some modifications were improper, this does not change the final results.

The corresponding author was candid and cooperative throughout the process but in light of the above issues, which raise concerns about the reliability of the reported results and data, the *PLOS Neglected Tropical Diseases* Editors retract this article.

AVAS, RCM, LHMM, EC, RP and FNM agreed with retraction and AVAS and FNM apologize for the issues with the published article. AVAS, RP and FNM stand by the article’s findings. FBF and AAM could not be reached.
